# Reverse Genetics of Orthoflaviviruses: Strategies for Constructing Functional or Infectious cDNA

**DOI:** 10.1002/jmv.71007

**Published:** 2026-06-10

**Authors:** Young‐Min Lee

**Affiliations:** ^1^ Department of Animal, Dairy, and Veterinary Sciences, College of Agriculture and Natural Resources Utah State University Logan Utah USA

**Keywords:** flavivirus, functional cDNA, infectious cDNA, orthoflavivirus, reverse genetics

## Abstract

Reverse genetics (RG) systems have transformed RNA virus research by enabling the recovery of recombinant viruses entirely from cloned cDNA. These platforms allow precise, hypothesis‐driven manipulation of viral genomes and support systematic phenotypic analyses in vitro and in vivo. Orthoflaviviruses, a genus that includes numerous zoonotic arboviruses associated with neurological and visceral disease, present distinct challenges for RG development. RNA‐launched systems, which rely on in vitro transcription of infectious RNA from a full‐length functional cDNA clone, have long been the standard approach. However, the technical demands of RNA synthesis and handling have increased interest in DNA‐launched systems that initiate virus recovery directly from a full‐length infectious cDNA clone. Despite these advances, constructing genetically stable full‐length orthoflavivirus cDNA clones remains difficult because certain genomic regions are unstable or toxic during bacterial propagation. To address these obstacles, conventional bacteria‐based cloning has been supplemented with bacteria‐free strategies such as long‐distance reverse transcription–PCR, circular polymerase extension reaction, Gibson isothermal assembly, and infectious subgenomic amplicons. This commentary summarizes the key features, strengths, and limitations of current RNA‐ and DNA‐launched RG systems for orthoflaviviruses, with emphasis on the technical challenges unique to this group. Together, these perspectives provide a framework for selecting and implementing RG approaches tailored to orthoflaviviruses.

1

The emergence of molecular biology in the 1970s marked a turning point in RNA virus research, driven largely by the development of reverse genetics (RG) systems. These systems enabled the cloning of viral genomic RNA into cDNA, its stable maintenance in bacterial plasmids, and the recovery of recombinant viruses from cloned cDNA [1]. The first successful application of RG was reported in 1978 for the positive‐strand RNA coliphage Qβ [2], followed in 1981 by the rescue of poliovirus from cloned cDNA [3]. This approach, now known as DNA‐launched RG, relies on intracellular transcription of viral cDNA to initiate replication. Limitations in transcription initiation accuracy and transcript processing fidelity subsequently motivated the development of RNA‐launched RG, in which full‐length viral cDNA serves as the template for in vitro synthesis of infectious RNA. The first RNA‐launched system was established in 1984 for brome mosaic virus, a positive‐strand RNA plant virus [4]. Since then, RNA‐launched systems have been widely adopted for many positive‐strand RNA viruses, including members of the *Orthoflavivirus* genus within the *Flaviviridae* family [5].

Orthoflaviviruses, formerly classified as flaviviruses, are primarily zoonotic arboviruses transmitted by mosquitoes or ticks. This group includes several clinically important pathogens such as Japanese encephalitis virus (JEV), West Nile virus (WNV), Zika virus (ZIKV), dengue virus (DENV), yellow fever virus (YFV), and tick‐borne encephalitis virus (TBEV) [6]. These viruses are associated with severe neurologic and visceral disease and continue to pose a global health threat [7]. Orthoflaviviruses are enveloped viruses with a single‐stranded, positive‐sense RNA genome of approximately 11 kb. The genome contains a 5′ cap structure but lacks a 3′ poly(A) tail. It encodes a single long open reading frame flanked by short noncoding regions. Translation produces a polyprotein that is cleaved into three structural proteins (C, prM, and E) and seven nonstructural proteins (NS1, NS2A, NS2B, NS3, NS4A, NS4B, and NS5) [8, 9]. A major milestone in orthoflavivirus research was the establishment of the first RG system for YFV in 1989 [10]. This RNA‐launched system used in vitro run‐off transcription of infectious RNA from a full‐length cDNA template assembled by in vitro ligation. This achievement paved the way for the development of both RNA‐ and DNA‐launched systems for many orthoflaviviruses (Figure 1). This commentary focuses on the key technical challenges associated with orthoflavivirus cDNA instability or toxicity, compares available cDNA cloning and assembly strategies, and highlights recent technological innovations, including two recent studies published in this journal.

**Figure 1:**
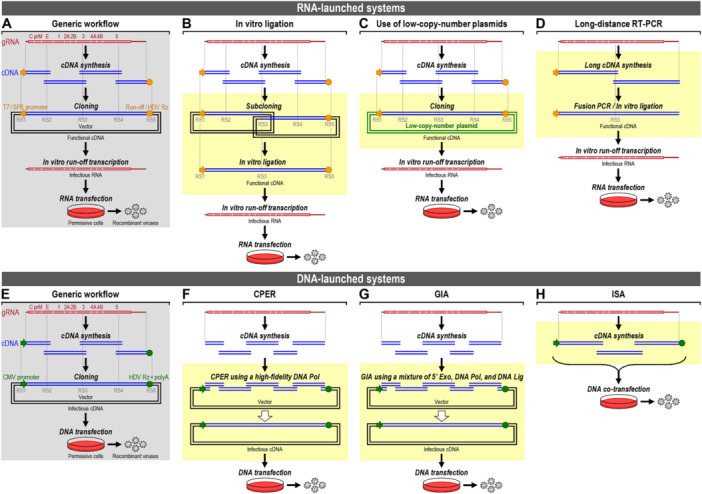
Strategies for generating functional or infectious orthoflavivirus cDNAs. (A–D) RNA‐launched systems. (A) Generic workflow illustrating four main steps: (i) synthesis of overlapping cDNA fragments representing the viral genomic RNA (gRNA, which contains a single long open reading frame encoding 10 viral proteins flanked by 5′ and 3′ noncoding regions); (ii) assembly of these fragments into a full‐length cDNA by cloning into a vector through restriction sites (RS1 to RS5); (iii) production of RNA transcripts from the assembled cDNA clone by in vitro run‐off transcription; and (iv) recovery of recombinant viruses from permissive cells transfected with the in vitro‐produced RNA transcript. The viral genome sequence is typically flanked at the 5′ end by a phage promoter such as T7 or SP6 and at the 3′ end by either a unique restriction site for run‐off transcription (Run‐off) or a hepatitis delta virus ribozyme (HDV Rz). (B–D) Three representative RNA‐launched systems with distinct features: (B) In vitro ligation, in which the viral genome is subcloned as two overlapping cDNA fragments, each maintained in a vector, and ligated in vitro using a unique restriction enzyme to generate a full‐length functional cDNA template for in vitro run‐off transcription. (C) Use of low‐copy‐number plasmids, which supports assembly and maintenance of the full‐length functional cDNA while reducing the intracellular burden associated with toxic viral sequences. (D) Long‐distance RT‐PCR, in which the full‐length functional cDNA template is generated by fusion PCR or by in vitro ligation of two long overlapping cDNA fragments representing the viral genome. (E–H) DNA‐launched systems. (E) Generic workflow illustrating three main steps: (*a*) synthesis of overlapping cDNA fragments representing the viral gRNA; (*b*) assembly of these fragments into a full‐length cDNA by cloning into a vector through restriction sites (RS1 to RS5); and (*c*) recovery of recombinant viruses from permissive cells transfected with the assembled cDNA clone. The viral genome sequence is typically flanked at the 5′ end by a cytomegalovirus (CMV) promoter and at the 3′ end by an HDV ribozyme (HDV Rz) followed by a polyadenylation signal (polyA). (F–H) Three representative DNA‐launched systems with distinct features: (F) Circular polymerase extension reaction (CPER), which assembles a full‐length infectious cDNA from overlapping fragments in a vector by using a high‐fidelity DNA polymerase to extend overlapping regions and generate a continuous circular DNA molecule without ligase. (G) Gibson isothermal assembly (GIA), which joins overlapping cDNA fragments into a full‐length infectious cDNA in a vector using a mixture of 5′ exonuclease (Exo), DNA polymerase (Pol), and DNA ligase (Lig). (H) Infectious subgenomic amplicons (ISA), which enable rapid virus recovery by co‐transfecting permissive cells with overlapping cDNA fragments that span the full viral genome. More detailed descriptions of each system are provided in the main text.

As one of the two principal strategies, RNA‐launched RG systems take advantage of the inherent infectivity of viral genomic RNA, which initiates autonomous replication in the cytoplasm once introduced into host cells [11]. A major advantage of this approach is its ability to closely mimic natural post‐entry events by bypassing viral entry and directly delivering genomic RNA to the cytoplasm, where viral protein synthesis, RNA replication, particle assembly, and release occur (Figure 1A). Among orthoflaviviruses, the first RNA‐launched system was established for YFV [10], followed by systems for JEV, WNV, ZIKV, DENV, and TBEV [12, 13]. Early systems for YFV, JEV, and DENV used a two‐plasmid strategy in which the viral genome was divided into two cDNA fragments that were ligated in vitro using a single restriction enzyme to generate a full‐length cDNA template (Figure 1B) [10, 14, 15]. A four‐plasmid strategy, in which four cDNA fragments were assembled through defined restriction sites, has also been used for DENV and ZIKV to produce full‐length templates suitable for in vitro transcription [16, 17, 18, 19]. These templates were flanked upstream by a strong phage promoter such as T7 or SP6 and downstream either by a unique restriction site to allow run‐off transcription or by a hepatitis delta virus (HDV) ribozyme to ensure precise 3′‐end formation.

Among orthoflaviviruses, JEV has been particularly difficult to clone as a full‐length cDNA in bacterial systems because of intrinsic instability or toxicity across high‐, medium‐, and low‐copy‐number plasmids [14, 20, 21, 22]. Our group and others identified the prM–E gene segment of JEV as especially problematic. When cloned into high‐copy‐number plasmids, this region consistently accumulated nonsense mutations during bacterial propagation, resulting in premature stop codons [14, 23]. Similar challenges have been addressed in many orthoflaviviruses, including JEV, WNV, ZIKV, DENV, YFV, and TBEV, by using low‐copy‐number plasmids to reduce the intracellular burden of toxic viral sequences (Figure 1C) [24, 25, 26, 27, 28, 29, 30, 31, 32, 33, 34, 35, 36, 37, 38, 39, 40]. In particular, bacterial artificial chromosomes (BACs) have been successfully applied to several orthoflaviviruses [23, 41, 42, 43, 44, 45, 46, 47, 48]. BACs are derived from the *Escherichia coli* fertility factor and were originally designed to accommodate large DNA inserts up to approximately 350 kb [49, 50]. They have proven effective for cloning full‐length cDNAs of many positive‐strand RNA viruses with genome sizes ranging from approximately 10 to 32 kb [51, 52]. Their major advantage is the ability to maintain genetic stability, although their low copy number limits DNA yield and their large size presents practical challenges [53]. In addition to plasmids and BACs, a cosmid vector has also been used to clone the full‐length cDNA of JEV [54].

Beyond vector selection, several additional strategies have been developed to address the instability or toxicity of full‐length orthoflavivirus cDNAs in *E. coli* [12, 13]. First, silent mutations can be introduced into toxic regions to reduce cryptic bacterial promoter activity. This approach has been effective for JEV, DENV, and ZIKV [55, 56, 57, 58]. Second, introducing an intron, such as a modified group II self‐splicing intron, into toxic regions disrupts cryptic promoter activity and reduces cDNA instability or toxicity. This strategy has been successfully used for ZIKV [59]. Similarly, insertion of a short linker containing multiple stop codons has stabilized DENV cDNA [60]. Third, inserting a tandem repeat sequence upstream of the viral genome suppresses cryptic promoter activity in downstream viral sequences and stabilizes full‐length cDNA. This method has been applied to JEV and DENV [61]. Fourth, a cloning vector derived from the linear double‐stranded DNA genome of coliphage N15 [62], maintained at low copy number and engineered with transcriptional terminators flanking the cloning site, reduces transcriptional interference and enhances the stability of full‐length ZIKV cDNA [63]. Fifth, different *E. coli* strains have been used to generate full‐length cDNA clones for orthoflaviviruses such as DENV and TBEV, likely by reducing selective pressures and genetic alterations [60, 64, 65, 66, 67]. Yeast has also been used to assemble full‐length DENV cDNAs through homologous recombination using either a yeast artificial chromosome or a yeast–*E. coli* shuttle vector [68, 69, 70]. Sixth, lowering culture temperature and shortening culture duration can improve cDNA stability by reducing replication stress on plasmids carrying unstable or toxic inserts [22, 23, 39, 71, 72]. Together, these strategies provide effective solutions to the long‐standing instability and toxicity challenges associated with full‐length orthoflavivirus cDNAs in bacterial systems and have enabled the successful construction of full‐length functional cDNA clones.

Alongside strategies to overcome viral cDNA instability or toxicity, two major technological advances have significantly improved the efficiency and feasibility of generating full‐length functional cDNA clones for positive‐strand RNA viruses, including orthoflaviviruses [53]. First, advances in enzymology, particularly the development of thermostable, processive reverse transcriptases and high‐fidelity DNA polymerases, have enabled accurate long‐range reverse transcription–PCR (RT‐PCR) [73]. In orthoflaviviruses, these enzymes allow the synthesis of two or three overlapping long cDNA amplicons spanning the entire approximately 11 kb genome, reducing the complexity and error rate associated with traditional multi‐fragment cloning. This capability facilitates the cloning of full‐length viral cDNA into a single plasmid. Second, improvements in in vitro transcription systems and RNA transfection protocols have simplified virus recovery [74, 75]. Infectious RNA transcribed in vitro from a full‐length cDNA clone can be directly introduced into host cells by electroporation, producing high‐titer recombinant viruses, typically 10^5^ to 10^6^ infectious particles per mL, within a few days. Despite these advances, constructing genetically stable full‐length cDNA clones in bacteria for RNA‐launched systems remains technically demanding and requires substantial labor, time, and expertise. As a result, bacteria‐free alternatives have emerged. In one approach, infectious RNA is synthesized from a full‐length cDNA template generated by fusion PCR or by ligating two long overlapping cDNA amplicons using a unique restriction site, eliminating the need for bacterial cloning (Figure 1D) [24, 76]. This method was demonstrated for TBEV, where high‐fidelity PCR using a pair of DNA polymerases, one with proofreading activity, produced two overlapping cDNA segments representing the viral genome. An SP6 promoter was appended to the 5′ end of the full‐length cDNA template to enable in vitro run‐off transcription of infectious RNA.

Compared with RNA‐launched systems, DNA‐launched RG systems enable the recovery of recombinant viruses directly from a full‐length infectious cDNA clone introduced into host cells (Figure 1E) [11]. In this approach, the full‐length orthoflaviviral cDNA is typically placed under the control of a cytomegalovirus (CMV) promoter, transcribed by cellular RNA polymerase II, and processed at the 3′ end by an HDV ribozyme followed by a polyadenylation signal, using vector systems similar to those employed for RNA‐launched systems [46, 77, 78, 79, 80, 81, 82, 83]. This strategy eliminates the need for RNA synthesis and handling. However, as with RNA‐launched systems, constructing genetically stable full‐length infectious cDNA clones remains challenging because specific genomic regions are unstable or toxic during bacterial propagation. To address these issues, several approaches have been used, similar to those applied in RNA‐launched systems, including the introduction of silent mutations into toxic regions [84], the insertion of one or two introns into the viral genome [46, 82, 85, 86, 87, 88, 89], modification of the CMV promoter to reduce its activity in bacteria [20], and insertion of a tandem repeat sequence upstream of the promoter‐derived viral genome [61, 83].

To more directly overcome these limitations, conventional bacteria‐based cloning has evolved into three main bacteria‐free methods [13]. The first two, circular polymerase extension reaction (CPER) and Gibson isothermal assembly (GIA), are conceptually similar. CPER assembles full‐length viral cDNA from multiple overlapping RT‐PCR amplicons in the correct order within a vector; during thermal cycling, a high‐fidelity DNA polymerase extends the overlapping regions, generating a continuous circular DNA molecule without the need for ligase (Figure 1F) [90]. CPER is simple and efficient for assembling two or three fragments, but its efficiency decreases as fragment number increases, and it lacks exonuclease activity. This method was first applied to construct an infectious cDNA clone of WNV and was later extended to ZIKV, JEV, TBEV, and the insect‐specific Parramatta River virus [91, 92, 93, 94, 95]. A recent study by Park and colleagues, published in this journal, described the construction of a full‐length infectious JEV cDNA clone in a BAC using the In‐Fusion cloning technique, which, although bacteria‐based, is ligation‐independent and conceptually similar to CPER [96]. It uses the 3′‐to‐5′ exonuclease activity of a DNA polymerase derived from Vaccinia virus to generate single‐stranded 5′ overhangs that anneal precisely at homologous regions between fragments. After transformation into *E. coli*, bacterial repair machinery seals the nicks. In a related approach, GIA uses a one‐pot, isothermal reaction to join multiple overlapping RT‐PCR products. It employs three enzymes: a 5′ exonuclease to generate single‐stranded overhangs, a DNA polymerase to fill gaps, and a DNA ligase to seal nicks (Figure 1G) [97]. GIA enables efficient assembly of up to ten or more fragments into a linear or circular DNA construct, although it requires specialized enzyme mixes that increase procedural complexity and cost. This technique was first used to construct infectious DENV cDNA clones [98]. For ZIKV, GIA has recently been applied to join two cDNA fragments encompassing the full‐length genome, flanked by a 5′ T7 promoter and a 3′ HDV ribozyme, enabling in vitro synthesis of the viral genome [99].

The third bacteria‐free method, infectious subgenomic amplicons (ISA), represents a distinct and highly simplified strategy for rapid virus recovery [100]. In this approach, permissive cells are co‐transfected with overlapping subgenomic RT‐PCR amplicons, typically three, that span the full viral genome (Figure 1H). Each fragment can be cloned into a bacterial plasmid for long‐term storage and used as a template for clonal PCR amplification. The terminal fragments are flanked by a 5′ CMV promoter and a 3′ HDV ribozyme followed by a polyadenylation signal. Intracellular recombination assembles the full‐length viral cDNA, from which viral RNA is transcribed and replicated. ISA is particularly advantageous for positive‐strand RNA viruses with large genomes. However, its efficiency depends on precise fragment design and the recombination capacity of the host cell, which may limit its reliability for viruses with low recombination rates or complex genome structures. ISA has been successfully applied to multiple orthoflaviviruses, including JEV, WNV, ZIKV, YFV, DENV, and TBEV [101, 102, 103]. In a recent article published in this journal, Singh and colleagues validated a modified ISA strategy for synthesizing toxic cDNA fragments from JEV and WNV [104]. For WNV, the study subdivided a long toxic C‐to‐NS1 coding region, which is unsuitable for stable bacterial cloning, into four shorter overlapping subfragments less than 1.8 kb each. This modification enabled rapid and efficient production of synthetic virus stocks without bacterial propagation. The work demonstrated that ISA provides a streamlined and effective method for generating recombinant JEV and WNV, which is particularly valuable for accelerating research during outbreak scenarios. Collectively, CPER, GIA, and ISA have significantly advanced the generation of full‐length infectious cDNAs for positive‐strand RNA viruses, including orthoflaviviruses, by mitigating issues of cDNA instability and toxicity. Beyond the construction of full‐length functional or infectious cDNAs, the performance of an RG system ultimately depends on its ability to generate infectious virus efficiently. Several factors influence virus recovery, including the choice of RNA or DNA transfection method, optimization of transfection parameters, selection of permissive cell lines, and post‐transfection culture conditions [100, 105, 106].

In summary, RG systems have transformed orthoflavivirus research by enabling precise manipulation of viral genomes. Built on functional or infectious cDNAs, these systems, including multiple platforms developed for JEV [14, 23, 33, 96, 104, 107, 108] whose full‐length cDNA is highly unstable and toxic in bacterial hosts, have advanced studies of viral replication, pathogenesis, and antiviral strategies. Two major types of systems exist, RNA‐launched and DNA‐launched, each with distinct strengths and limitations. The choice between them depends on experimental goals. RNA‐launched systems, particularly those based on full‐length functional cDNA clones, are well suited for analyzing *cis*‐acting RNA elements required for replication, whereas DNA‐launched systems, especially ISA‐based approaches, are ideal for rapid virus recovery. Continued innovation is expected to broaden the impact of these systems. Advances in synthetic genomics are reducing the cost and time required for de novo DNA synthesis, making routine construction of full‐length viral cDNAs possible without bacterial propagation. Emerging bacteria‐free assembly methods may support rapid generation of large libraries of viral variants for functional screening. Integrating RG with high‐throughput sequencing [109, 110] and single‐cell and spatial transcriptomics [111, 112, 113] will deepen our understanding of genome replication, gene expression, genetic diversity, viral evolution, virus–cell interactions, and host responses. In parallel, replication‐competent but propagation‐incompetent replicon platforms may provide safer tools for studying viral replication and developing antiviral drugs [114, 115, 116]. Finally, RG is poised to accelerate rational vaccine design, including codon‐modified and chimeric vaccines [117, 118, 119, 120]. Together, these advances highlight the growing potential of RG systems to drive both fundamental discovery and translational progress against emerging and re‐emerging orthoflaviviruses.

## Author Contributions

The author conducted the literature review, drafted the manuscript, and performed critical revisions.

## Conflicts of Interest

The author declares no conflicts of interest.

## Data Availability

The author has nothing to report.
